# T cells, more than antibodies, may prevent symptoms developing from respiratory syncytial virus infections in older adults

**DOI:** 10.3389/fimmu.2023.1260146

**Published:** 2023-10-13

**Authors:** Bruno Salaun, Jonathan De Smedt, Charlotte Vernhes, Annick Moureau, Deniz Öner, Arangassery Rosemary Bastian, Michel Janssens, Sunita Balla-Jhagjhoorsingh, Jeroen Aerssens, Christophe Lambert, Samuel Coenen, Christopher C. Butler, Simon B. Drysdale, Joanne G. Wildenbeest, Andrew J. Pollard, Peter J. M. Openshaw, Louis Bont

**Affiliations:** ^1^ GSK, Rue de l’Institut, Rixensart, Belgium; ^2^ Sanofi, Lyon, France; ^3^ Biomarkers Infectious Diseases, Janssen Pharmaceutica NV, Beerse, Belgium; ^4^ Janssen Vaccines & Prevention B.V., Leiden, Netherlands; ^5^ Centre for General Practice, Department of Family Medicine and Population Health (FAMPOP), University of Antwerp, Antwerp, Belgium; ^6^ Laboratory of Medical Microbiology, Vaccine & Infectious Disease Institute (VAXINFECTIO), University of Antwerp, Antwerp, Belgium; ^7^ Nuffield Department of Primary Care Health Sciences, University of Oxford, Oxford, United Kingdom; ^8^ Oxford Vaccine Group, Department of Paediatrics, University of Oxford, and the National Institute for Health and care Research (NIHR) Oxford Biomedical Research Centre, Oxford, United Kingdom; ^9^ Department of Paediatric Infectious Diseases and Immunology, Wilhelmina Children’s Hospital, University Medical Center Utrecht, Utrecht, Netherlands; ^10^ National Heart and Lung Institute , Imperial College London, London, United Kingdom; ^11^ ReSViNET Foundation, Julius Clinical, Zeist, Netherlands

**Keywords:** respiratory syncytial virus, cell-mediated immunity, correlate of protection, interferon-gamma, T-cell memory, CD4+ T cell, antibody function, machine learning

## Abstract

**Introduction:**

The immune mechanisms supporting partial protection from reinfection and disease by the respiratory syncytial virus (RSV) have not been fully characterized. In older adults, symptoms are typically mild but can be serious in patients with comorbidities when the infection extends to the lower respiratory tract.

**Methods:**

This study formed part of the RESCEU older-adults prospective-cohort study in Northern Europe (2017–2019; NCT03621930) in which a thousand participants were followed over an RSV season. Peripheral-blood samples (taken pre-season, post-season, during illness and convalescence) were analyzed from participants who (i) had a symptomatic acute respiratory tract infection by RSV (RSV-ARTI; N=35) or (ii) asymptomatic RSV infection (RSV-Asymptomatic; N=16). These analyses included evaluations of antibody (Fc-mediated–) functional features and cell-mediated immunity, in which univariate and machine-learning (ML) models were used to explore differences between groups.

**Results:**

Pre–RSV-season peripheral-blood biomarkers were predictive of symptomatic RSV infection. T-cell data were more predictive than functional antibody data (area under receiver operating characteristic curve [AUROC] for the models were 99% and 76%, respectively). The pre-RSV season T-cell phenotypes which were selected by the ML modelling and which were more frequent in RSV-Asymptomatic group than in the RSV-ARTI group, coincided with prominent phenotypes identified during convalescence from RSV-ARTI (e.g., IFN-γ+, TNF-α+ and CD40L+ for CD4+, and IFN-γ+ and 4-1BB+ for CD8+).

**Conclusion:**

The evaluation and statistical modelling of numerous immunological parameters over the RSV season suggests a primary role of cellular immunity in preventing symptomatic RSV infections in older adults.

## 1 Introduction

In older adults, respiratory syncytial virus (RSV) infections typically give rise to mild upper respiratory coryzal symptoms but can lead to serious or life-threatening pneumonia (1–5). Previous infections provide partial protection from reinfection and disease (6), but this immunity is usually transient (7). The RESCEU older-adults prospective-cohort study was designed to further understand the relationship between natural immunity and the epidemiology of RSV in older adults. A thousand participants aged at least 60 years were recruited in Northern Europe and followed over an RSV season between 2017 and 2019, and provided samples of peripheral-blood and nasopharyngeal swabs (8). The RSV infection rate was estimated at 4.2 and 7.2% during the 2017-2018 and 2018-2019 RSV-seasons, respectively (9).

The analyses reported thus far by RESCEU found an association between the susceptibility to RSV acute respiratory tract infection (RSV-ARTI) and levels of certain types of antibodies (Öner DRESCEU investigators, 2023^1^). We have shown that susceptibility to RSV-ARTI is related to: (i) the pre–RSV-season serum level of pre-F–specific IgGs; (ii) the pre–RSV-season mucosal level of pre-F–specific IgAs (but not IgGs); and (iii) the pre–RSV-season serum level of RSV G-protein–specific IgGs. By contrast, no relationships were found between symptomatic and asymptomatic infections and levels of RSV-specific antibodies, including neutralizing antibodies (Öner DRESCEU investigators, 2023^1^). Elsewhere, other recent studies have identified functional features in antibodies beyond neutralization that protect against RSV-challenge; including features reinforced by vaccination (10, 11).

The potential importance of T-cell responses has also been highlighted by RSV-challenge studies (6, 12–14), which show enhanced susceptibility to RSV-ARTI with age and an associated with a decline in RSV F-specific IFN-γ–producing T cells in peripheral blood. However, it is not known to what extent T-cell recognition contributes to defense against natural symptomatic infection.

The three objectives of this study were (i) to identify relationships between RSV-ARTI and T-cell responses to RSV, (ii) to identify if the susceptibility to RSV-ARTI can be predicted by T-cell or functional antibody data prior to the RSV-season; and (iii) to assess whether combining T-cell data with functional antibody data would improve predicting susceptibility to RSV-ARTI.

## 2 Materials and methods

### 2.1 Study design and demographics

The samples were obtained from the RESCEU older adult study (ClinicalTrials.gov: NCT03621930), which was a multi-country, multi-center, longitudinal, prospective, and observational cohort study (8, Öner DRESCEU investigators, 2023^1^). Overall, 1040 community-dwelling adults aged over 60 years were recruited before the 2017-2018 or 2018-2019 RSV season (1st of October to 1st May) and followed during the subsequent season. The individuals were from the general community in The Netherlands, Belgium, and the United Kingdom. The median age was 75 years and 54% were female, and there were no remarkable differences in the demographic characteristics between participants who had RSV-ARTI and those who did not (median ages were75.5 and 76.0 years, respectively (8, Öner DRESCEU investigators, 2023^1^). In the present study, two groups of individuals were analyzed and were identified from within the entire RESCEU older adult population. These individuals (i) had ARTI symptoms and were RSV infected (i.e., positive molecular point-of-care test and qPCR; RSV-ARTI group; N=35) or (ii) had no ARTI and were RSV infected (RSV-Asymptomatic group; N=16) based on having RSV-specific antibody titers (RSV A2 neutralizing, or preF or postF binding) that increased ≥4-fold at the end of the RSV-season from pre–RSV-season baseline (Öner DRESCEU investigators, 2023^1^). Hence blood samples were collected from both groups pre– and post–RSV-season visits (Visits 1 and 3); and for the RSV-ARTI group, when the subject presented with ARTI (Visit 2) and ~2-weeks later during convalescence (Visit 2c). However, not all blood samples were evaluable. For the comparison with the RSV-Asymptomatic group, only a subset of the RSV-ARTI group was used (the RSV-ARTI 4X subset N=11), in which subjects also had RSV-specific antibody titers that increased ≥4-fold over the RSV-season (up to Visit 3) from the pre–RSV-season baseline, to remove a potential bias from differences in antibody levels.

The RESCEU study was approved by institutional review boards in Belgium, the Netherlands and the United Kingdom, and participants gave informed consent before taking part in the study (8, Öner DRESCEU investigators, 2023^1^).

### 2.2 CD4+ and CD8+ T cells frequencies in peripheral-blood samples

CD4+ and CD8+ T-cell frequencies were measured by the *in vitro* stimulation of PBMC cultures and staining for immune markers (15). Briefly, PBMCs were stimulated with (i) RSV-A lysate, (ii) RSV-B lysate; or pools of peptides (15mers overlapping by 11) spanning the coding sequences for (iii) RSV-A F, (iv) M2-1, or (v) N; or PBMCs were not stimulated (cultured with medium only) for background measurements. RSV lysates (commercially available from Zeptometrix, Buffalo, NY) were propagated in the Vero cell line, purified using sucrose density gradient, ultracentrifugation, and disrupted in the presence of 0.5% Triton X-100 non-ionic detergent/0.6 M KCl and heat inactivated) For PBMC stimulation, virus lysates were used at a final concentration of 5 µg/mL and peptides at 1.25 µg/mL. PBMCs were stimulated for approximately 18 hours, with the last 16 hours including Brefeldin A in the culture medium to promote intracellular accumulation of cytokines. The analysis focused exclusively on live cells, and the staining of CD3, CD4 and CD8 was used to identify T cells. The staining for markers 4-1BB, CD40L, IL-2, IL-13, IL-17, IFN-γ, and TNF-α was used to indicate antigen specificity and phenotype. Polypositive T cells (CD4+ or CD8+) were defined as T cells expressing at least two immune markers, including at least one cytokine. From prior assay validation, the lower limit of quantification (LLoQ) was defined as 590 polypositive CD4+ (or CD8+) T cells per million CD4+ (or CD8+) T cells, and the limit of blank (LoB) was set at 310 polypositive CD4+ (or CD8+) T cells per million CD4+ (or CD8+) T cells. These values came from assay validation: the LLoQ equaled the lowest frequency detectable with a coefficient of variation below 50%; and the LoB was descriptive and not used as a cut-off in the analysis and represents the 95th percentile of the distribution of background-subtracted frequencies when only medium is used to stimulate cells. Linear regression was used to analyze correlations between T-cell frequencies above assay LLoQ when samples were stimulated by RSV-A and RSV-B lysates, respectively.

### 2.3 Evaluation of Fc mediated antibody functionalities

The system serology assessment was performed at SeromYx Systems (SeromYx Systems, Inc., Cambridge, MA 02139, USA) using the preF antigen for defining target specificity (16). In addition to FcR binding, isotyping, and subclassing, functional assays were performed for antibody-dependent cellular phagocytosis (ADCP); cell-type specific antibody-dependent phagocytosis involving basophils (ADBP), dendritic cells (ADDCP), eosinophils (ADEP), and neutrophils (ADNP); and antibody-dependent cellular cytotoxicity (ADCC), NK cell activation (ADNKA) and complement deposition (ADCD). For more detail on the functional-assay methods, see references (17, 18) and **Supplementary Methods**.

### 2.4 Differential abundance analysis

For T-cell frequencies, Poisson regression models were fitted on log-transformed data, in which the respective total numbers of CD4+ or CD8+ T cells were used as offsets.

For the antibody titers, two-sided Student’s t tests were used to identify significant differences between groups.

For the different T-cell phenotypes and functional antibody types, respectively, the ratios between means of symptomatic data points and asymptomatic data points were ranked by absolute magnitude. For the different T-cell phenotypes, the adjusted *P*-values ≤0.05 set the threshold for those ratios to be included in the ranking. For the functional antibody types, unadjusted *P*-values were used to provide a less stringent threshold for the analysis than adjusted *P*-values. *P*-values were adjusted using Benjamini-Hochberg multiple-test correction and are described in the relevant table (T cells) and figure (antibodies).

### 2.5 General machine learning strategy

In general, the machine learning (ML) strategy was composed of two parts, i.e., the optimization and the assessment (See **Supplementary Methods**). The optimization step was used for feature selection, as well as for hyperparameter tuning for all ML models considered (i.e., logistic regression [LR], random forests [RF], support vector machines [SVM], K-nearest neighbors [KNN], and gradient-boosting classifiers [GBC]). All ML-modelling was performed using the Scikit-Learn framework in Python.

The procedures for excluding values from the ML modelling are described in the **Supplementary Methods**.

## 3 Results

### 3.1 The T-cell response to natural RSV infection

**Figure 1:**
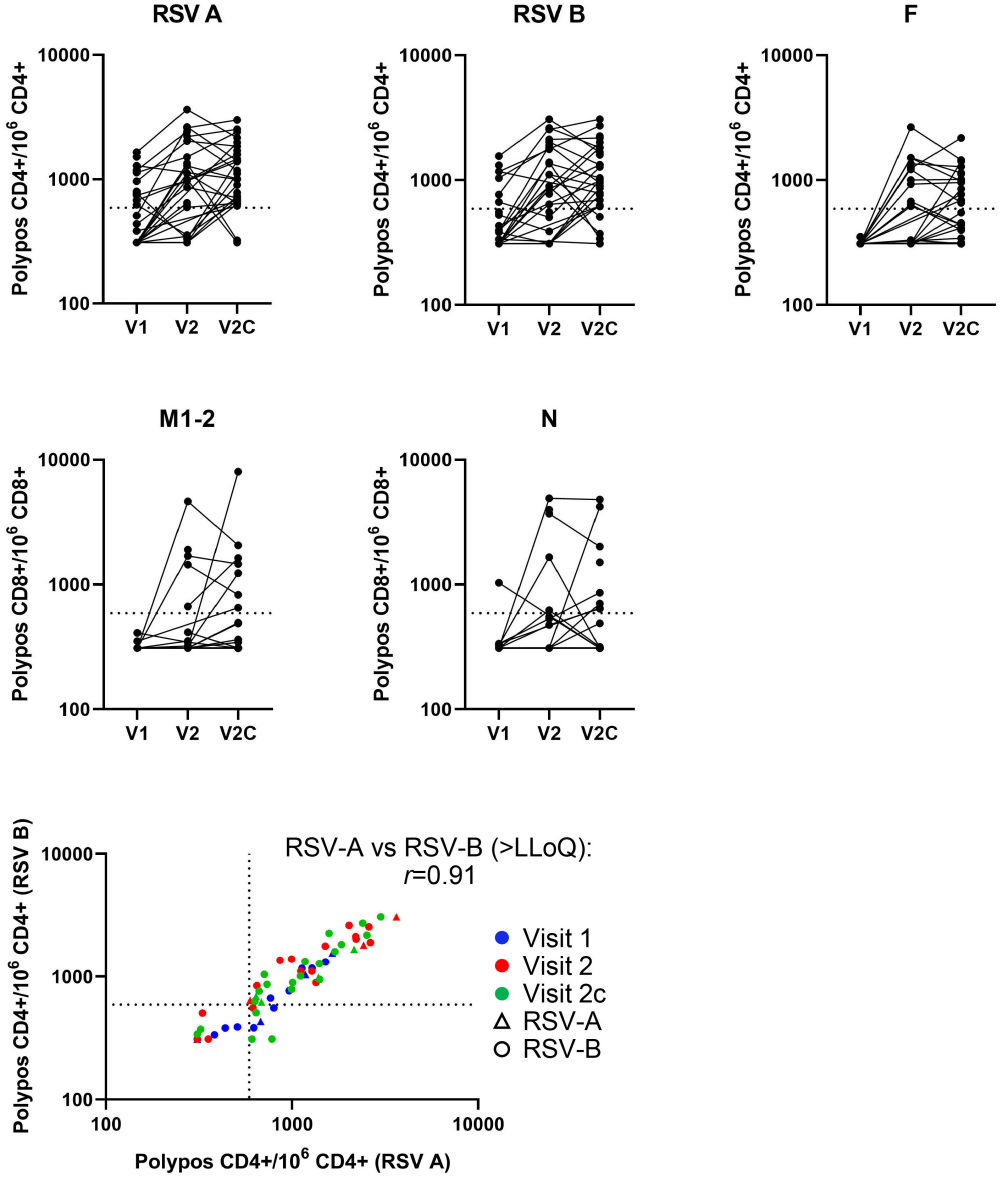
T-cell kinetics change over the course of an RSV symptomatic infection. Background subtracted T-cell frequencies by subject (in the RSV-ARTI group) and by visit (i.e., time point). **(A)** T-cell frequencies are shown at pre-RSV season (Visit 1 [V1]; N=27), from when symptomatic RSV-ARTI was reported (Visit 2 [V2]; N=24), and during convalescence about 2 weeks later (Visit 2c [V2c]; N=29). RSV-A–specific, RSV-B–specific and F-specific CD4+ T-cell frequencies (per million CD4+ T cells), and M2-1–specific and N-specific CD8+ T-cell frequencies (per million CD8+ T cells) are shown for the polypositive phenotype (i.e., positive staining for at least two immune markers including at least one cytokine among 4-1BB, CD40L, IL-2, IL- 13, IL-17, IFN-γ, and TNF-α). **(B)** RSV-A–specific polypositive CD4+ T-cell frequencies plotted against RSV-B–specific polypositive CD4+ T-cell frequencies for individual subjects over the three visits, and by the type of RSV infection. All values are shown, including those below the lower limit of quantification (LLoQ; 590; horizontal and vertical dotted lines). Values below the limit of blank (LoB; 310) were set to 310. The *r* coefficient describes a correlation between T-cell frequencies >LLoQ stimulated by RSV-A and RSV-B lysates, respectively.

T-cell responses to RSV antigens were followed over the RSV season in the study subjects who developed a symptomatic, laboratory-confirmed RSV infection (i.e., in the RSV-ARTI group). In the pre–RSV-season period (Visit 1), the frequencies of CD4+ (but not CD8+) T cells specific for epitopes in whole-virus lysates were above the lower limit of quantification (LLoQ) in peripheral-blood samples of some RSV-ARTI subjects (**Table 1**, **Figure 1A**). These included 11/27 (41%) subjects for RSV A and 7/27 (26%) subjects for RSV B. CD4+ T-cell frequencies above the LLoQ for antigens F, M2-1 or N were not identified in any subject suggesting that the RSV-A–specific and RSV-B–specific CD4+ T cells detected pre-RSV season predominantly targeted other antigens. However, in two subjects only, CD8+ T-cell frequencies above the LLoQ were identified either for antigens F or N, respectively. By contrast, CD8+ T-cell frequencies above the LLoQ were not identified after stimulation with RSV lysates probably because the nature of the assay was not geared to effective presentation of lysate fragments on HLA-A/B/C (MHC-I).

**Table 1:** Social Demographic Characteristics.

Percentage of subjects (number of subjects / total number of subjects with evaluable samples) in the RSV-ARTI group with T-cell frequencies above LLoQ with respect to antigen stimulation in PBMC assay					
	RSV-A lysate	RSV-B lysate	F peptides	M2-1 peptides	N peptides
**CD4+**					
Visit 1	41 (11/27)	26 (7/27)	0 (0/27)	0 (0/27)	0 (0/27)
Visit 2	75 (18/24)	71 (17/24)	50 (12/24)	4 (1/24)	30 (7/23)
Visit 2c	93 (27/29)	83 (24/29)	46 (13/28)	4 (1/28)	14 (4/28)
**CD8+**					
Visit 1	0 (0/22)	0 (0/22)	4 (1/23)	0 (0/23)	4 (1/24)
Visit 2	5 (1/22)	5 (1/21)	4 (1/23)	23 (5/22)	24 (5/21)
Visit 2c	0 (0/25)	4 (1/24)	13 (3/24)	29 (7/24)	33 (8/24)

LLoQ, lower limit of quantification; Visit 1, pre–RSV-season visit; Visit 2, visit when RSV-ARTI was reported; and Visit 2c, convalescence visit about 2 weeks after reporting RSV-ARTI.

Symptomatic RSV infection was associated with the activation of T cells (**Table 1**, **Figure 1A**). At the time RSV-ARTI was reported (Visit 2) and in comparison to pre-RSV season (Visit 1), higher proportions of subjects displayed RSV-specific CD4+ T-cell frequencies above the LLoQ (RSV-A–specific, 18/24 (75%) versus 11/27 (41%); and RSV-B–specific, 17/24 [71%] versus 7/27 [26%]). At the convalescence time point about 2 weeks later (Visit 2c), these proportions of subjects were generally higher again (RSV-A–specific, 27/29 [93%]; and RSV-B–specific, 24/29 [83%]). At the same time point, higher proportions of subjects had CD4+ T-cell frequencies above the LLoQ that were specific for F (12/24 [50%] then 13/28 [46%]) than for N (7/23 [30%] then 4/28 [14%]), or M2-1 (1/24 [4%] then 1/28 [4%]), suggesting F epitopes were immunodominant for CD4+ T cells over those from the other RSV antigens tested. Moreover, RSV-A–specific and RSV-B–specific CD4+ T-cell frequencies above the LLoQ by individual subjects were positively correlated (*r*=0.91), irrespective of whether the infections were by RSV-A or RSV-B, suggesting that the epitopes that were recognized by those CD4+ T cells appeared to be conserved between RSV A and RSV B (**Figure 1B**). Although lower than with CD4+ T cells, the proportion of subjects with RSV-specific CD8+ T-cell frequencies above the LLoQ also increased from pre-RSV season (Visit 1) to the time of reporting RSV-ARTI (Visit 2) and 2 weeks after that (Visit 2c), notably for M2-1-specific CD8+ T cells (0/23[0%] to 5/22 [23%] and then 7/24 [29%]), and for N-specific CD8+ T cells (1/24 [4%] to 5/21 [24%] and then 8/24 [33%]; **Table 1**, **Figure 1A**).

**Figure 2:**
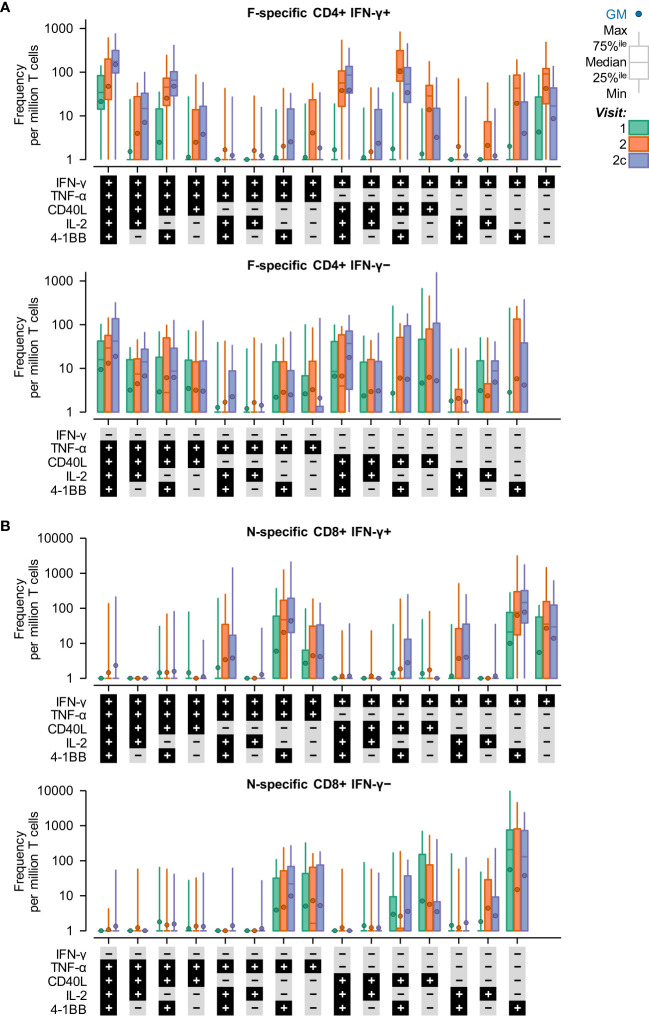
IFN-γ expression dominates the T-cell response to symptomatic RSV infection. Background subtracted T-cell frequencies by phenotype and time point in the RSV-ARTI group. T-cell frequencies are shown at pre-RSV season (Visit 1; CD4+, N=27; CD8+, N=24), from when symptomatic RSV-ARTI was reported (Visit 2; CD4+, N=24; CD8+, N=21), and during convalescence about 2 weeks later (Visit 2c; CD4+, N=28; CD8+, N=24). Phenotype was determined by positive staining for at least one immune markers among 4-1BB, CD40L, IL-2, IL-13, IL-17, IFN-γ, and TNF-α. **(A)** F–specific CD4+ T-cell frequencies (per million CD4+ T cells) and **(B)** N–specific CD8+ T-cell frequencies (per million CD8+ T cells) are shown (all phenotypes were negative for IL-13 and IL-17). A histogram bar describes the median frequency, and the whiskers describe the 25th to 75th percentile interval. Frequencies below 1 were imputed the value of 1. All values are shown, including those below the lower limit of quantification (LLoQ) when analyzed at the polypositive level. 40L, CD40L; IL2, IL-2; TNF, TNF-α; IFN, IFN-γ; and 1BB, 4-1BB (CD137).

Symptomatic RSV infection was also associated with a transition in the phenotypes of RSV-specific (F-specific) CD4+ T cells and RSV-specific (N-specific) CD8+ T cells from effector to polyfunctional memory (**Figure 2**). Moreover, almost all T-cell phenotypes were double negative for IL-13 and IL-17 (hence, these markers are not shown in **Figure 2**). The prominent CD4+ T-cell phenotypes that peaked (in terms of median frequencies) when symptomatic RSV infection was reported (Visit 2) were dominated by IFN-γ production, including (i) IFN-γ+, (ii) IFN-γ+ plus CD40L+ or 4-1BB+, and (iii) IFN-γ+, CD40L+ and 4-1BB+. The prominent CD4+ T-cell phenotypes that peaked at the convalescence time point (Visit 2c) included (i) IFN-γ+, TNF-α+, CD40L+, IL-2+ and 4-1BB+, and (ii) CD40L+ plus double or triple positive for IL-2, IFN-γ, or TNF-α. The prominent CD8+ T-cell phenotypes at Visit 2 and Visit 2c were IFN-γ+ plus (i) 4-1BB+, and (ii) 4-1BB+ TNF-α.

### 3.2 Pre–RSV-season assessment of T-cell frequencies and susceptibility to symptomatic infection

Pre-RSV season, the proportions of subjects with RSV-A–specific and RSV-B–specific polypositive CD4+ T-cell frequencies above the LLoQ in the RSV-Asymptomatic group (N=12) were similar to those observed in the RSV-ARTI-4X subset of the RSV-ARTI group (N=11; **Supplementary Figure 1**). Also, RSV-A–specific and RSV-B–specific CD4+ T-cell frequencies above the LLoQ in individual subjects from both the RSV-Asymptomatic group and RSV-ARTI-4X subset were positively correlated (*r*=0.91). Therefore, the frequencies of RSV-specific polypositive CD4+ T cells or the degree of possible cross-reactivity to A and B types exhibited by those T cells, appeared not to be associated with protection against symptomatic RSV infection.

**Figure 3:**
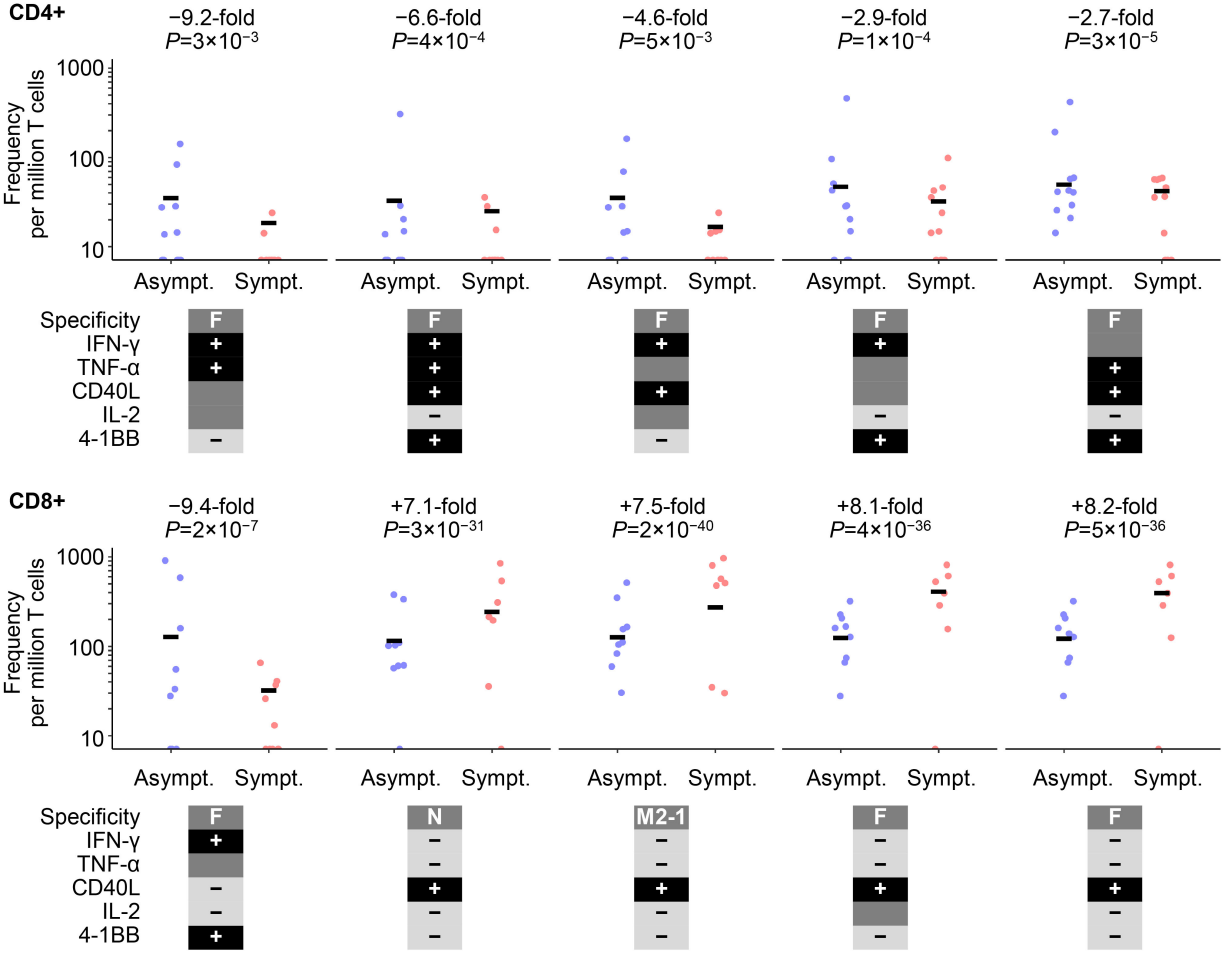
Cell-mediated–immunity (CMI) features associated with symptomatic RSV infection. Poisson regression analysis on all cell-mediated–immunity (CMI) features after data preprocessing identified eight CD4+ and 25 CD8+ T-cell populations that were differentially abundant between the RSV-Asymptomatic group (Asympt.) and the RSV-ARTI-4X subset (Sympt.; see **Supplementary Table 1**). The top five cell populations with the highest absolute fold differences are shown for **(A)** CD4+ T cells and **(B)** CD8+ T cells. Only subjects with T-cell data for CD4+ (N=11 and 11, respectively by group) or CD8+ (N=10 and 9, respectively by group) were used for the regression analysis. Fold differences were computed as the ratio of the means of values of symptomatic subjects and the values of asymptomatic subjects. Unadjusted *P*-values are also shown, although, adjusted *P*-values ≤0.05 set the threshold for those ratios to be included in the ranking (see **Supplementary Table 1**). RSV-ARTI 4X subset included those subjects in the RSV-ARTI group who had a ≥4-fold increase in RSV-specific antibody titers over the RSV season. 40L, CD40L; IL2, IL-2; TNF, TNF-α; IFN, IFN-γ; IL13, IL-13, IL17, IL-17; and 1BB, 4-1BB (CD137). Circular symbols describe individual values and horizontal lines describe means.

However, differences in RSV-specific CD4+ and CD8+ T-cell frequencies were identified pre-RSV season (Visit 1) between the RSV-Asymptomatic group and RSV-ARTI-4X subset, using a univariate Poisson regression analysis that considered all T-cell data (i.e., including all possible combinations of immune markers expressed, and irrespective of the polypositive frequency being below or above LLoQ; **Figure 3**). Overall, eight CD4+ phenotypes and 25 CD8+ phenotypes were significantly different between the RSV-Asymptomatic group (N=11 and 9, respectively) and the RSV-ARTI-4X subset (N=11 and 10, respectively; **Supplementary Table 1**), when using adjusted *P*-values ≤0.05 as the threshold. The highest fold differences with the top five ranked CD4+ phenotypes ranged from 9.2 to 2.7- fold higher frequencies in the RSV-Asymptomatic group than in the RSV-ARTI-4X subset. All five phenotypes were F-specific, dominated by IFN-γ production, and negative for IL-13 and IL-17. The highest fold differences with the top five ranked CD8+ phenotypes were first, a 9.4 higher frequency (F-specific and IFN-γ+, 4-1BB+), and then a range of 8.4 to 7.1 lower frequencies in the RSV-Asymptomatic group than in the RSV-ARTI-4X subset (F- or M2-1– or N-specific and generally positive for CD40L and negative for IFN-γ, TNF-α, IL-13 and IL-17). Hence the T-cell phenotypes that appeared higher in the RSV-Asymptomatic group than in the RSV-ARTI-4X subset corresponded to F-specific, effector/effector-memory phenotypes.

### 3.3 Pre–RSV-season assessment of functional antibodies and susceptibility to symptomatic infection

**Figure 4:**
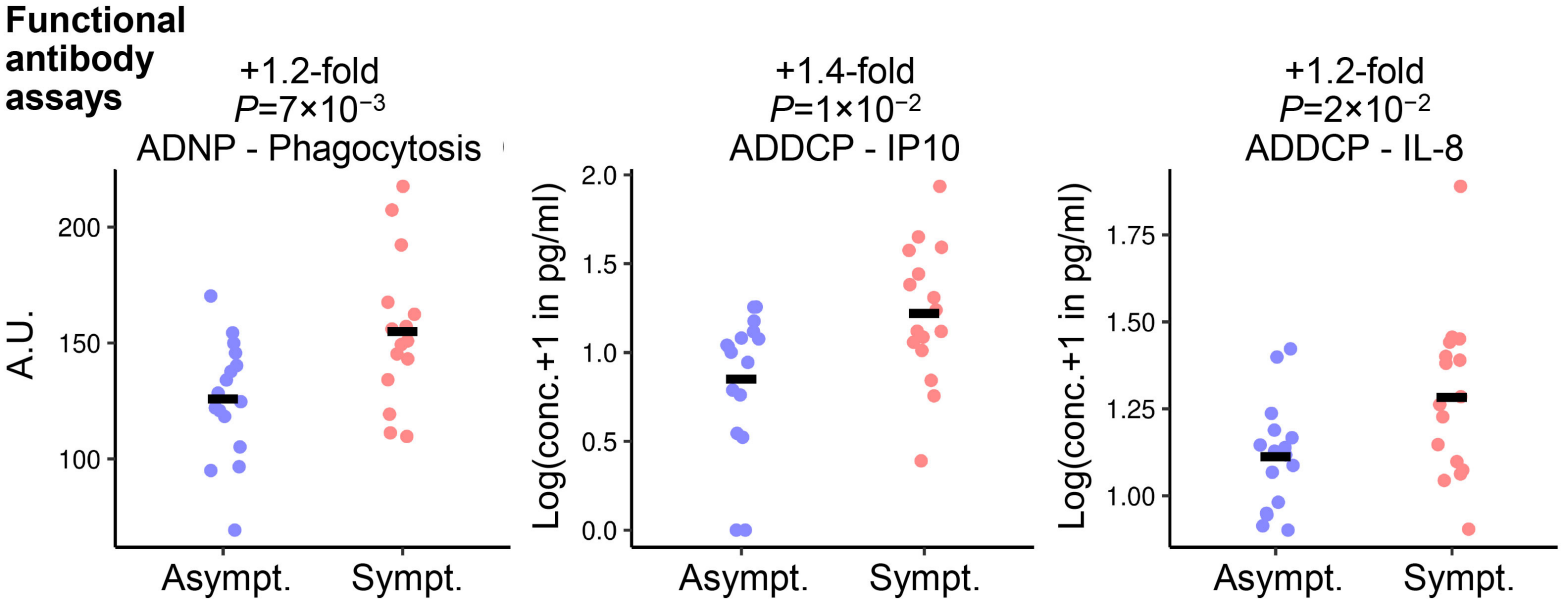
Functional antibody features associated with symptomatic RSV infection. Student’s t-test identified three functional antibody features differing between RSV-ARTI-4X group (Sympt.; N=16) and RSV-Asymptomatic group (Asympt. N=16). Fold differences were computed as the ratio of the means of values of symptomatic subjects and the values of asymptomatic subjects, and the unadjusted *P*-values ≤0.05 set the threshold for those ratios to be included in the ranking. The three function features are (i) ADP by DCs leading to IL-8 signaling (ADDCP - IL8); (ii) antibody-dependent phagocytosis (ADP) by DCs leading to IP-10 signaling (ADDCP - IP10); and (iii) ADP by neutrophils (ADNP - Phagocytosis). The adjusted *P*-values (using the Benjamini-Hochberg multiple-test correction) were (i) 0.2, (ii) 0.1, and (iii) 0.1. Circular symbols describe individual values and horizontal lines describe means.

Differences in serum levels of preF-specific antibodies classified by type and function between RSV-Asymptomatic groups and the RSV-ARTI-4X subset were also identified by univariate Student’s t testing (**Figure 4**, **Supplementary Table 2**), but only when using unadjusted *P*-values ≤0.05 as the threshold. Overall, three functional features appeared less prevalent in the RSV-Asymptomatic group (N=16) than in RSV-ARTI-4X subset (N=16). These included (i) antibody-dependent phagocytosis (ADP) by DCs leading to IP-10 signaling, (ii) ADP by neutrophils, and (iii) ADP by DCs leading to IL-8 signaling.

### 3.4 Predictive models of susceptibility to symptomatic disease based on T-cell and antibody data

**Figure 5:**
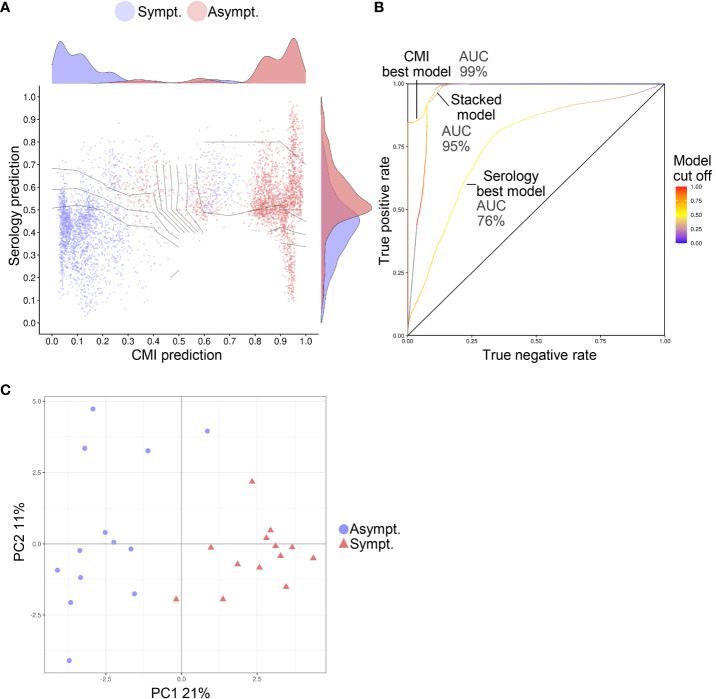
Predictive models of symptomatic RSV infection. The susceptibility to RSV ARTI could be predicted from samples collected prior to the RSV-season. **(A)** Graphic visualization of the predictions made by the machine learning (ML) models that were trained on T-cell data only (represented on x-axis) or on serology data only (represented on y-axis). Prediction values of 0 indicate predicted asymptomaticity, whereas values of 1 indicate predicted symptomaticity. The stacked ML model was trained on the output predictions of the former two ML models (represent by its decision boundaries [curved lines]). Predictions were made for each test set in a 200-times 5-fold crossvalidation. Hence, for each subject 200 predictions are shown. Only data of subjects with both T-cell data and antibody data were used for training and testing (N=26). **(B)** Receiver operating characteristic (ROC) curves show the performance of the unstacked and stacked ML models. True-positive and false-positive rates were computed on the predictions made in a 200-times 5-fold crossvalidation setting on the 26 subjects with both T-cell and functional antibody data. Areas under the ROC curves (AUCs) are shown for the best serology and CMI models and for the stacked model. **(C)** Principal component (PC) analysis on the features selected by the unstacked ML models shows separation between asymptomatic (Asympt.) and symptomatic (Sympt.) subjects (N=26) when plotted by PC1 and PC2. The percentage contribution of the PCs to the variation in the data are shown on the axis labels.

Using ML, models were identified that could predict protection against symptomatic RSV infection from pre–RSV-season data (**Figure 5**). In these models, features that discriminated between the RSV-Asymptomatic and RSV-ARTI-4X subset were selected (i.e., T-cell specificities and phenotypes or antibody functions). A stacked model was then trained on the output predictions of those models. The predictive capacity of the best T-cell–data model was greater than the best antibody-data model, as indicated by the greater area under the receiver operating characteristic curve (AUROC; 99% versus 76%) and the improved ability to separate data points from the two groups. The stacked ML model had a similar predictive capacity (AUROC, 95%) to that of the best T-cell–data model. Moreover, a principal component analysis (PCA) on the data associated with the features selected by the T-cell–data models and the antibody-data models (**Supplementary Table 2**), clearly discriminated between the two groups in a plot of the first two principal components (**Figure 5**).

## 4 Discussion

We monitored RSV-specific T cells in peripheral-blood samples from the RESCEU older-adult cohort (8, Öner DRESCEU investigators, 2023^1^) over the course of a natural RSV infection and identified relationships between symptomatic RSV infection and T-cell responses to RSV prior to the RSV-season. Our study also suggested that the susceptibility of developing respiratory-tract symptoms from an RSV infection could be predicted by pre-RSV–season T-cell data. Although functional antibody data could predict to a lesser degree, the susceptibility of developing symptoms from an RSV infection, combining functional antibody data with T-cell data did not further improve the predictive value of the models built on T-cell data alone. The T-cell phenotypes that appeared higher pre-RSV season in those whose subsequent RSV infection was asymptomatic rather than symptomatic overlapped with prominent and potential memory phenotypes identified during convalescence from RSV-ARTI (e.g., CD4+, CD40L+, IFN-γ+, TNF-α+, and CD8+ IFN-γ+, 4-1BB+). The production of IFN-γ was also a prominent feature of the CD4+ T-cell phenotypes mobilized during the infection. To our knowledge, this is the first report describing a shift in functional profiles of T cells over the course of a natural RSV infection. T-cell engagement during the course of infection suggested a role of the cellular adaptive immunity in controlling infection, in agreement with observations from experimental RSV challenge models (13).

The interpretations of the results should be cautioned by the limitations of the study, in that the population analyzed was small, samples were only obtained from peripheral-blood and not the mucosa (12), and the statistical models were not validated on independent data sets. Moreover, as described before in older adults (14), the frequencies of RSV-specific T cells in the peripheral blood of older adults were typically low, notably for particular phenotypic subsets. Furthermore, our modelling analysis was data-driven and hence dependent on the nature of the assays used to generate the data (i.e. RSV-specific frequencies of T-cell subsets for the cell-mediated analysis, and the level of pre-F specific antibodies categorized by functional features). We recognize that assays focused on other functional aspects of T cells (degree of cytokine production, degranulation or exhaustion) or on antibody specificities for other antigens, may have identified additional categories in asymptomatic versus symptomatic infections. There are also other biases that may shape data interpretation. The selection criterion for asymptomatic cases used a 4-fold increase in antibody titers over the RSV season, and in the modelling, this criterion was also applied to the symptomatic cases. This tended to exclude individuals with higher pre-RSV–season titers (Öner DRESCEU investigators, 2023^1^). However, when lower thresholds (~1.5-fold) had been used for determining inclusion into the asymptomatic groups, no differences had been identified between asymptomatic and symptomatic groups for F-binding or neutralizing antibody titers (Öner DRESCEU investigators, 2023^1^). A method for detecting asymptomatic infections that was independent of measuring immune markers, such as PCR, would have required too frequent sampling throughout the entire follow up in all participants, and it was therefore not implemented.

Although the CD4+ T-cell response to symptomatic RSV infection appeared dominated by effector cells geared towards IFN-γ production, the prevalent subpopulations detected at convalescence were more polyfunctional, with four to five immune markers expressed, consistent with an effector-memory phenotype. Interestingly, the fully polyfunctional phenotype (expressing 5 immune markers) was the main phenotype detected pre-RSV season. Presumably, this pre–RSV-season phenotype reflected previous exposure(s) to RSV, given that F-specific antibodies had been detected in all study participants (Öner DRESCEU investigators, 2023^1^).

Pre-RSV season, the predominant epitopes recognized by the CD4+ T cells appeared not to have been within RSV F, but possibly within RSV G protein in the RSV lysates because G protein has been described as an immunodominant CD4+ T-cell antigen (12). Nevertheless, and although below the LLoQ pre-RSV season, F-specific polypositive CD4+ T cells were detected. Some polypositive subsets (mainly IFN-γ–producing T cells) were less frequent in subjects who went on to have symptomatic RSV infections than in subjects who went on to have asymptomatic infections. Similarly, F-specific effector CD8+ T cells (IFN-γ–producing 4-1BB+ T cells) were less frequent, again pointing towards to a role of F-specific T-cell immunity in the control of natural RSV infection. These results were comparable to what has been observed for another respiratory virus, influenza, where lower frequencies of polyfunctional CD4+ T cells and IFN-γ+ CD8+ T cells were identified in adults who went on to develop symptoms after infections (19). By contrast, CD8+ T cells expressing CD40L (but none of the other markers tested), were more frequent in subjects who went on to have symptomatic RSV infections than in subjects who went on to have asymptomatic infections. The nature and role of this CD8+ T-cell subset was unclear. The CD40L+ phenotype is unusual for CD8+ T cells and is usually restricted to CD4+ T cells. CD8+ T cells have been described in the CD8+ memory compartment of LCMV infected mice, but these cells expressed additional cytokines upon stimulation (20). Therefore, additional investigations are needed to elucidate their function and association with susceptibility to symptomatic infection.

Although not as strong as with T cells, an association was identified from statistical modelling between pre–RSV-season serum-antibody functional features and the susceptibility of developing symptoms from an RSV infection. However, no association was identified with the differential abundance analysis for an individual antibody functional feature when the P-value was adjusted for multiple comparisons. One of the three highest ranked features by (positive) fold difference included the induction of neutrophil phagocytosis, which coincides with the linkage identified with RSV-challenge, between neutrophilic inflammation and susceptibility to symptomatic RSV infection (21). By contrast, antibody-mediated cell phagocytosis has been a feature associated with preventing infection in RSV-challenge studies; a feature that was reinforced with a preF-coding–adenovirus vaccine candidate (10). One possible explanation for the different observations is that the RSV-challenge studies evaluated the prevention of infection (i.e., the absence of live virus in nasal swabs) (10, 11, 22) whereas our study evaluated (indirectly) the prevention of respiratory-tract symptoms from infection because of the likelihood that RSV-asymptomatic subjects were infected (hence the ≥4-fold increase of RSV-specific antibodies). Therefore, our predictive modelling was skewed towards identifying factors that prevented the progression of infection to symptomatic disease rather than factors (typically antibody-mediated) that prevented or restricted the initial infection *per se*.

In conclusion, the evaluation and statistical modelling of numerous immunological parameters over the RSV season suggests a primary role of cellular immunity in preventing symptoms from developing with RSV infections in older adults. Hence, the identification of certain F-specific T-cell effector-memory populations associated with prevention of symptomatic infection, provides useful markers for understanding natural immunity to RSV in older age and assessing the induction (and persistence) of T-cell responses to candidate RSV vaccines, some of which were nearing commercial release.

## Data availability statement

The raw data supporting the conclusions of this article will be made available by the authors, without undue reservation.

## Ethics statement

The RESCEU study was approved by institutional review boards in Belgium, the Netherlands and the United Kingdom, and participants gave informed consent before taking part in the study [8, 10]. The studies were conducted in accordance with the local legislation and institutional requirements. The participants provided their written informed consent to participate in this study.

## Author contributions

BS: Writing – original draft, Writing – review & editing. JD: Writing – original draft, Writing – review & editing. CV: Writing – original draft, Writing – review & editing. AM: Writing – original draft, Writing – review & editing. DÖ: Writing – original draft, Writing – review & editing. AB: Writing – original draft, Writing – review & editing. MJ: Writing – original draft, Writing – review & editing. SB-J: Writing – original draft, Writing – review & editing. JA: Writing – original draft, Writing – review & editing. CL: Writing – original draft, Writing – review & editing. SC: Writing – review & editing. CB: Writing – review & editing. SD: Writing – original draft, Writing – review & editing. JW: Writing – original draft, Writing – review & editing. AP: Writing – original draft, Writing – review & editing. PO: Writing – original draft, Writing – review & editing. LB: Writing – original draft, Writing – review & editing.

## Footnotes

1 Correlates of protection from rsv infection in community-dwelling older adults.

## Group members of RESCEU investigators

Michael Abram, AstraZeneca; Philippe Beutels, Vaccine & Infectious Disease Institute, University of Antwerp; Harry Campbell, University of Edinburgh; Thea K. Fischer, Statens Serum Institut; Scott Gallichan, Sanofi; Carlo Giaquinto, PENTA Foundation; Terho Heikkinen, University of Turku and Turku University Hospital; Veena Kumar, Novavax; Federico Martinon-Torres, Servicio Galego de Saude; Adam Meijer, National Institute for Public Health and the Environment; Eva Molero, Team-It Research; Harish Nair, University of Edinburgh; Kena Swanson, Pfizer; and Maarten van den Berge, University of Groningen.
