# Supplementary material for: T cells, more than antibodies, may prevent symptoms developing from respiratory syncytial virus infections in older adults
Source: Front Immunol. 2023 Oct 13;14:1260146. doi: 10.3389/fimmu.2023.1260146 (PMC10627235; doi:10.3389/fimmu.2023.1260146)
Supplement: Supplementary file 1 [file DataSheet_1.pdf]

Supplementary Material

T cells, more than antibodies, may prevent symptoms developing from respiratory syncytial virus (RSV) infections in older adults

Bruno Salaun*, Jonathan De Smedt, Charlotte Vernhes, Annick Moureau, Deniz Öner, Arangassery Rosemary Bastian, Michel Janssens, Sunita Balla-Jhagjhoorsingh, Jeroen Aerssens, Christophe Lambert, Samuel Coenen, Christopher C. Butler, Simon B. Drysdale, Joanne G. Wildenbeest, Andrew J. Pollard, Peter J. M. Openshaw, Louis Bont on behalf of the RESCEU Investigators.

*** Correspondence:** Bruno Salaun: bruno.x.salaun@gsk.com

1 Supplementary Methods

1.1 Serology data preprocessing

Flow-cytometer–based assays for (i) antibody dependent phagocytosis (ADP) by dendritic cells (DCs) leading to IL-10 signaling and (ii) ADP by DCs leading to TNF-α signaling, returned values marked as invalid response or quantity not sufficient from 32/32 and 31/31 subjects, respectively, and therefore no background values were subtracted. All other measurements with values below the limits of background (LoBs) were replaced by zero. After inspection of the distributions of each assay's measurements, a log10 (x+1) transformation was applied on all measurements expressed in mean fluorescence intensity (MFI) or in concentrations (pg/ml). Subsequently, hierarchical clustering (with the distance being computed using Pearson correlation) was applied in order to remove features with strong linear relationships (which may negatively affect feature selection and machine-learning [ML] performance). Upon manual inspection, the hierarchical clustering tree was cut at height = 0.35 to group correlated features. Eigenvalues were computed for each feature group. Within each group, a proxy feature was defined as the feature with the highest Pearson correlation with the respective group's eigenvalue. Thus, the selected proxy features were the most representative for all features in their respective feature groups. Only proxy features were considered in downstream analyses.

1.2 Cell-mediated immunity data preprocessing

For each subject and within each group (RSV-ARTI-4X or RSV-Asymptomatic), background measurements of cell numbers (i.e., T cells not exposed to any antigen) were subtracted from all other cell counts. All resulting negative cell counts were replaced by zero. The data from 3/29 subjects were entirely excluded because both CD4+ and CD8+ T-cell counts were negative. For the data from the remaining subjects, missing values were imputed using the median of the log-transformed abundance values (i.e., cells-per-million + 1) of data from samples stimulated with the same antigen from subjects in the same group). Additional variables were computed for both CD4+ T cells and CD8+ T cells based on phenotype (i.e., combinations of positive and negative detection of immune markers, CD40L, IFN-γ, IL-2, and TNF-α. CD4/CD8 ratios were also computed. Overall, 21871 features were defined for T-cell data. Next, cell populations defined by a given feature were removed from analysis on condition that for all samples (i) values were zero; (ii) sparsity >70%; or (3) variance <1. Multicollinearity between the remaining features was assessed through hierarchical clustering (complete linkage) wherein the distance metric was computed using Pearson correlation. Upon manual inspection, the resulting dendrogram was cut at height = 0.3, such that each cluster contained various features defining the same cell population. Within each cluster, the most precise feature was retained as the defining (proxy) feature for subsequent analysis, resulting in a final set of 458 features for downstream analysis.

1.3 Machine-learning (ML) optimization

Data (stratified by group; RSV-ARTI-4X or RSV-Asymptomatic) were randomly split into one set (90% of the data) for optimization and one hold-out set (10% of the data) for assessment of the feature selection and hyperparameter tuning steps. To avoid overfitting, a nested crossvalidation (CV) approach was used, wherein the outer CV (20 times repeated 5-fold CV, allowed performance evaluation of a set of selected features and hyperparameters on unseen data, whereas the inner CV (5-fold CV) aided avoiding overfitting of the feature selection and hyperparameter tuning procedure. All training data sets were scaled prior to model training.

Feature selection was performed using either lasso logistic regression, random forests, or Boruta (1). Features and hyperparameters with the highest area under the receiver operating characteristic (AUROC) on inner CV test sets were selected and used for prediction on the outer CV test sets. Features and hyperparameters that were selected most frequently across all outer CV folds were considered optimal and retained for the assessment part. The three feature selection methods were used independently and each of the three resulting feature sets were used for modelling using each of the five ML models. Hence there were 15 independent ML strategies. Only when looking at the final results in each analysis, we evaluated which combination of feature selection methods and ML models had the best performance.

1.4 ML assessment

First, the initial data hold-out set (which had not been used for the optimization step) was used to assess the performance of ML models with the selected features and hyperparameters. Next, the selected features and hyperparameters were used to fit ML models using the entire data set. Specifically, 100-times repeated 10-fold CV (stratified by group; RSV-ARTI-4X or RSV-Asymptomatic) was applied to the entire data set. Training data were used to fit the ML models with the selected features and hyperparameters.

The CMI and serology data sets were analyzed with a stacked ML strategy wherein the output predictions of both CMI and serology models were used as input data. Data were split randomly 90%:10%: in which the 10% portion represented the hold-out test data set. The features were limited to those that were selected in the ML optimization steps on CMI data only or serology data only. A nested CV approach was used (20 times repeated 5-fold CV (stratified by group; RSV-ARTI-4X or RSV-Asymptomatic) such that only subjects represented in both CMI and serology data were divided into the 5 CV folds. Next, models, with hyperparameters optimized for either CMI data only or serology data only, were used to train on both the respective CV training data sets and on data of subjects that were not represented in both data sets. Subsequently, predictions made based on the CMI data and predictions made based on the serology data were used as inputs to stacked ML models. Again, LR, RF, SVM, KNN, and GBC classifier models were optimized. No further feature selection was considered for the stacked ML models. ML assessment for the stacked ML strategy consisted of 200-times repeated 5-fold CV.

2 Reference

1. Kursa MB, Rudnicki WR. Feature Selection with the Boruta Package. *Journal of Statistical Software* (2010) 36(11):1 - 13. doi: 10.18637/jss.v036.i11.

Supplementary Table 1: Features selected from all T-cell data that differentiate between the RSV-Asymptomatic Group and the RSV-ARTI-4X subset

**Feature**		**Fold difference**	**P-value**	**Adjusted**
**Type and functional phenotype**	**Antigen**	**RSV-ARTI-4X vs RSV-Asymptomatic Minus = lower; plus = greater**		**P-value***
CD4+ (IL13−IL17−)				
1BB−IFN+TNF+	F	−9.2	3×10^{-03}	2×10^{-02}
40L+IL2−TNF+IFN+1BB+	F	−6. 6	4×10^{-04}	4×10^{-03}
1BB^{-}40L+IFN+	F	−4.6	5×10^{-03}	3×10^{-02}
1BB+IFN+IL2−	F	−2.9	1×10^{-04}	1×10^{-03}
1BB+40L+IL2−TNF+	F	−2.7	3×10^{-05}	3×10^{-04}
1BB+40L+IFN+IL2−	N	−2.5	5×10^{-03}	3×10^{-02}
1BB+IFN+	F	−2.4	7×10^{-10}	9×10^{-09}
40L+IL2+TNF+IFN+1BB+	F	−2.0	5×10^{-05}	4×10^{-04}
CD8+ (IL13−IL17−)				
1BB+40L−IFN+IL2−	F	−9.4	2×10^{-07}	2×10^{-06}
40L+IL2−TNF−IFN−1BB−	F	+8.2	5×10^{-36}	1×10^{-34}
1BB−40L+IFN−TNF−	F	+8.1	4×10^{-36}	1×10^{-34}
40L+IL2−TNF−IFN−1BB−	M2-1	+7.5	2×10^{-40}	6×10^{-39}
40L+IL2−TNF−IFN−1BB−	N	+7.1	3×10^{-31}	7×10^{-30}
40L+IFN−IL2−TNF−	M2-1	+6.9	9×10^{-42}	3×10^{-40}
40L+IL2−TNF−IFN−1BB−	Lysate A	+6.2	3×10^{-54}	3×10^{-52}
40L+IFN−IL2−	N	+5.4	2×10^{-30}	4×10^{-29}
40L−IL2−TNF−IFN+1BB−	F	−5.0	4×10^{-03}	2×10^{-02}
40L+IL2−TNF−IFN−1BB−	Lysate B	+5.0	1×10^{-54}	2×10^{-52}
40L−IL2−TNF+IFN+1BB+	M2-1	−4.5	4×10^{-04}	4×10^{-03}
40L−IL2−TNF−IFN+1BB+	M2-1	−4.0	6×10^{-05}	5×10^{-04}
40L−IFN+TNF+	M2-1	−3.6	9×10^{-06}	9×10^{-05}
40L−IFN+IL2−	M2-1	−2.9	7×10^{-08}	7×10^{-07}
1BB−40L−IFN+IL2−	F	−2.9	7×10^{-03}	4×10^{-02}
At least 2	F	−2.7	7×10^{-08}	8×10^{-07}
At least 2	M2-1	−2.7	8×10^{-10}	9×10^{-09}
40L−IL2−TNF−IFN+1BB−	Lysate B	−2.7	5×10^{-03}	3×10^{-02}
1BB+40L−IL2−TNF+	M2-1	−2.6	3×10^{-04}	3×10^{-03}
40L−IFN+IL2−TNF−	M2-1	−2.4	3×10^{-04}	3×10^{-03}
40L−IL2−TNF−IFN−1BB+	F	−2.3	2×10^{-66}	9×10^{-64}
1BB+40L−IFN−IL2−	F	−2.3	1×10^{-65}	3×10^{-63}
1BB+40L−IL2−TNF+	F	−2.2	3×10^{-03}	2×10^{-02}
40L−IFN+IL2−TNF−	Lysate A	−2.2	6×10^{-03}	4×10^{-02}
1BB+40L−IL2−TNF−	M2-1	−2.0	3×10^{-52}	3×10^{-50}

PBMC stimulations were performed with peptides for RSV antigens, F, M2-1, and N, and whole virus lysates from RSV-A (Lysate A) and RSV-B (Lysate B). The RSV-ARTI-4X subset included those subjects in the RSV-ARTI group who had a ≥4-fold increase in RSV-specific antibody titers over the RSV season. *The adjusted *P*-values were calculated using the Benjamini-Hochberg multiple-test correction. Abbreviations: 40L, CD40L; IL2, IL-2; TNF, TNF-α; IFN, IFN-γ; IL13, IL-13, IL17, IL-17; and 1BB, 4-1BB (CD137).

Supplementary Table 2: Features selected in stacked ML model for predicting symptomatic RSV infection.

Feature			Significant
Type	Functional phenotype	Antigen	
CD4	1BB-IFN+IL13-IL17-TNF+	F	Yes
CD4	1BB-40L+IFN+IL13-IL17-IL2-	Lysate B	No
CD4	40L-IL2-TNF+IFN-IL13-IL17-1BB-	M2-1	No
CD4	40L-IL2-TNF-IFN-IL13+IL17-1BB-	Lysate B	No
CD4	1BB-40L+IFN+IL13-IL17-IL2-	N	No
CD4	1BB-IFN+IL13-IL17-TNF-	M2-1	No
CD4	40L-IL2+TNF-IFN-IL13-IL17-1BB-	Lysate B	No
CD4	40L+IL2-TNF-IFN+IL13-IL17-1BB-	Lysate B	No
CD4	40L+IL2+TNF+IFN-IL13-IL17-1BB-	N	No
CD4	1BB+40L+IL13-IL17-IL2+TNF+	N	No
CD4	40L+IL2+TNF-IFN+IL13-IL17-1BB+	Lysate A	No
CD4	1BB+40L+IL13-IL17-IL2-TNF+	M2-1	No
CD4	1BB-40L+IFN+IL13-IL17-	M2-1	No
CD4	40L+IL2+TNF-IFN-IL13-IL17-1BB+	Lysate B	No
CD4	1BB-IFN+IL13-IL17-IL2-	M2-1	No
CD4	40L+IL2+TNF+IFN-IL13-IL17-1BB+	N	No
CD4	40L+IFN-IL13-IL17-IL2+	F	No
CD4	1BB+40L+IFN-IL13-IL17-TNF+	M2-1	No
CD4	IL13-IL17-IL2+TNF+	M2-1	No
CD4	1BB+40L-IFN-IL13-IL17-TNF-	N	No
CD4	40L-IL2-TNF+IFN-IL13-IL17-1BB+	Lysate A	No
CD4	1BB-IFN-IL17+IL2-TNF-	F	No
CD4	1BB+40L+IL13-IL17-TNF+	M2-1	No
CD8	40L-IL2-TNF-IFN+IL13-IL17-1BB-	Lysate B	Yes
CD8	IFN-IL13-IL17-IL2-TNF+	F	No
CD8	40L-IL2-TNF+IFN+IL13-IL17-1BB+	N	No
CD8	1BB-IFN-IL13-IL17-IL2-TNF+	Lysate B	No
CD8	1BB+IFN-IL13-IL17-IL2+TNF-	Lysate A	No
CD8	1BB+40L-IL13-IL17-IL2+	Lysate A	No
CD8	1BB-40L-IL17-IL2+	M2-1	No
Ab	ADDCP_IL8_(1:50)	PreF	Yes
Ab	ADDCP_IP10_(1:50)	PreF	Yes
Ab	ADNP_PhagocytosisScore_(1:50)	PreF	Yes
Ab	IgSubclass_IgM_(1:100)	PreF	No
Ab	ADCD_C3_(1:20)	PreF	No
Ab	ADDCP_PhagocytosisScore_24hour_(1:50)	PreF	No
Ab	ADCC_Percent_lysis_(1:150)	PreF	No
Ab	ADCP_PhagocytosisScore_(1:600)	PreF	No

For identifying CD4+ and CD8+ T cells, PBMC stimulations were performed with peptides for RSV antigens F, M2-1, and N, and whole virus lysates from RSV-A (Lysate A) and RSV-B (Lysate B). The RSV-ARTI-4X subset included those subjects in the RSV-ARTI group who had a ≥4-fold increase in RSV-specific antibody titres over the RSV season. *No correction for multiple testing was made in assigning significance. Abbreviations: Ab, antibody; 40L, CD40L; IL2, IL-2; TNF, TNF-α; IFN, IFN-γ; IL13, IL-13, IL17, IL-17; and 1BB, 4-1BB (CD137).

Supplementary Figure 1: RSV-A–specific and RSV-B–specific CD4+ T-cell frequencies at the pre–RSV-season visit.

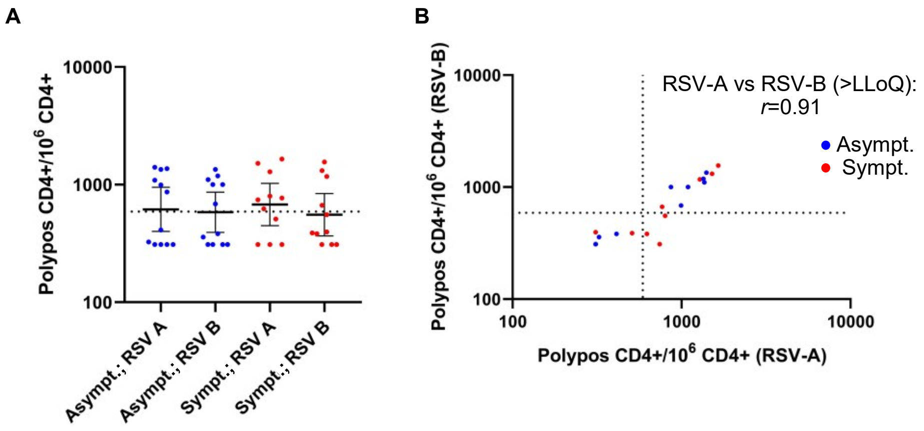

Background subtracted CD4+ T-cell frequencies by group (RSV-Asymptomatic group [N=12] versus the RSV-ARTI-4X subset [N=11]) at the pre–RSV-season visit. (A) RSV-A–specific and RSV-B–specific CD4+ T-cell frequencies (per million CD4+ T cells) are shown for the polypositive phenotype (i.e., positive staining for at least two immune markers (including at least one cytokine) among 4-1BB, CD40L, IL-2, IL-13, IL-17, IFN-γ, and TNF-α). Horizontal bars and whiskers represent geometric means and 95% confidence intervals. (B) RSV-A–specific CD4+ T-cell frequencies plotted against RSV-B–specific CD4+ T-cell frequencies for individual subjects. All values are shown, including those below the LLoQ value (horizontal and vertical dotted lines). Values below LoB (310) are set to 310. The RSV-ARTI-4X subset included those subjects in the RSV-ARTI group who had a ≥4-fold increase in RSV-specific antibody titres over the RSV season.
